# Targeted adult hearing loss screening in the UK: policy options for equitable implementation through health-service pathways

**DOI:** 10.3389/fpubh.2026.1904770

**Published:** 2026-07-13

**Authors:** Francesco Aletta

**Affiliations:** 1 Institute for Environmental Design and Engineering, Bartlett School of Environment Energy and Resources, University College London, London, United Kingdom; 2 Institute of Epidemiology and Healthcare, Faculty of Population Health Sciences, University College London, London, United Kingdom

**Keywords:** audiometry, dementia, hearing loss, NHS Health Check, screening

## Abstract

Adult hearing loss is common, disabling, and frequently under-detected, with help-seeking often delayed and unmet need unevenly distributed across populations. In the UK, adult hearing-loss screening is not currently recommended, and detection largely depends on self-recognition, primary-care presentation, and referral. This Policy Brief examines three policy options: maintaining the status quo, introducing age-based screening at 60, 65 and 70 years, and implementing targeted risk-based screening through existing health-service pathways for adults at elevated risk of cognitive decline. Appraisal against public health impact, feasibility, equity, and economic considerations suggests that targeted risk-based screening is the most immediately viable option. A phased pilot embedded in dementia, mild cognitive impairment, NHS Health Check, and audiology pathways could generate implementation evidence while improving equitable access to hearing care.

## 1 Introduction

Hearing loss is a very common and limiting condition in later life. Recent estimates show that, globally, 43.5 million years lived with disability (YLDs) can be attributed to hearing loss. This number has increased dramatically in the last three decades, and in the late 2010s' age-related hearing loss was the third largest source of YLDs and the first largest source for adults 70+ years of age (3). For this reason, the World Health Organization (WHO) has previously called for integration of “*people-centered ear and hearing care within national health plans for universal health coverage*” (1). In the UK context, 18 million adults are estimated to live with some degree of hearing loss (2). The case for adult hearing screening is also strengthened by demographic age shift: population aging is expected to increase the absolute number of people with hearing loss even where age-standardized prevalence remains relatively stable (3).

Unaddressed hearing loss can reduce access to spoken communication and situational awareness (via environmental auditory information), thus compromising daily functioning. It has also been linked to an increased risk of depression, cognitive decline and dementia (4). The consequences of non-identification are not only clinical: population evidence links adult hearing loss with economic hardship, including lower income and substantially higher odds of unemployment or underemployment, implying wider productivity losses and, by extension, reduced fiscal contribution (5). In later life, hearing impairment has also been associated with poorer physical functioning, incident disability and need for nursing care, while dementia-prevention evidence identifies hearing impairment as a modifiable risk factor, suggesting that delayed identification may increase downstream support needs for individuals, families and care systems (6). The Lancet Commission on dementia prevention, intervention and care identifies hearing impairment as a potentially modifiable midlife risk factor for dementia, supporting the case for earlier identification and management of hearing loss within healthy-aging and dementia-prevention strategies (7).

Despite effective treatment being available, only one in five people who could benefit from hearing aids currently have them, and help-seeking is often delayed by 10 to 12 years (2). The current UK system relies mainly on opportunistic identification through self-recognition, general practitioner (GP) presentation and referral, rather than systematic screening. This is problematic because self-reported hearing difficulty is not sufficiently sensitive to detect all clinically important hearing loss. In a large English study using the *English Longitudinal Study of Aging*, self-report missed a substantial proportion of objectively measured cases (8). The burden of hearing loss is also unevenly distributed at population level: recent UK evidence highlights a pronounced north-south divide, with age-adjusted prevalence among adults aged 71–80 years ranging from about 36% in South East England to 49% in the North East (9).

Notwithstanding, the policy environment is cautious: the UK National Screening Committee (UK NSC) currently does not recommend screening for hearing loss in adults, based on its latest review. On the other hand, the National Institute for Health and Care Excellence (NICE) guidance already recognizes hearing loss as an important comorbidity in adults with suspected or diagnosed dementia or mild cognitive impairment (MCI), and recommends audiology referral, with two-yearly reassessment where hearing loss has not previously been diagnosed (10).

These patterns strengthen the rationale for change because they suggest that the status quo may perpetuate delayed detection and widen inequalities. In this context, key stakeholders for national policy, clinical guidance, delivery and commissioning, and affected individuals are reported in Table 1.

**Table 1:** Stakeholders for the provision of adults' hearing loss screening in the UK.

Stakeholder	Impact	Interest
UK national screening committee	High	High
DHSC (as national policymakers)	High	High
NHS England/devolved administrations	High	High
ICBs/commissioners	High	High
Adult audiology providers	Medium	Medium
GPs/primary care teams	Medium	High
Local authorities (as NHS Health Check commissioners)	Medium	Medium
Older adults eligible for screening	Medium	High
People with MCI/dementia risk	Medium	High
Carers/families	Low	High
Patient and charity organizations	Low	High

## 2 Identification of policy options

### 2.1 Current policy (status quo)

As of 2026, universal screening for hearing loss in adults is not recommended in the UK. This is based on the most recently available evidence review published in 2021 by the UK National Screening Committee (UK NSC), which has the mandate within the Department of Health and Social Care for commissioning national screening programs. Rather than cost-effectiveness analysis, the UK NSC position is mostly driven by practical reasons: newer screening approaches, including smartphone-based tests, were not considered sufficiently accurate; although hearing aids would be the main intervention following detection, there is uncertainty about how willing people would be to use them; there is also no clear evidence that adults identified through screening would experience better health outcomes if they went on to use hearing aids; and, finally, it remains unclear how effectively hearing loss in adults is already being detected and managed across the UK (in the absence of a formal screening programme).

### 2.2 Option 1: targeted age-based screening

Acknowledging that universal screening for hearing loss in adults is not currently recommended, the first alternative policy option aims to introduce a one-stage NHS hearing screening offer for adults at ages 60, 65 and 70, using a brief pure-tone audiometric test designed to identify bilateral hearing loss at around 30 dB HL (i.e., hearing loss in decibels) (11). In practice, eligible adults would be identified from GP records, excluding those already under audiology care or already using hearing aids, and invited for screening at the relevant age points. For this option, an implementation issue may concern the reliability of eligibility data. Excluding people already using hearing aids is sensible to avoid unnecessary screening, but this depends on hearing-aid use and current audiology care being accurately coded in general practice records. Routine primary-care electronic records are powerful tools for population identification, but coding completeness can vary by practice, condition and recording purpose, and data-quality improvement often requires explicit feedback, training and validation processes (12, 13). Therefore, an age-based pilot should not rely solely on coded GP data to determine exclusion. Invitation systems should include a simple self-declaration route for people already using hearing aids or currently under audiology care. This would reduce inappropriate invitations while avoiding systematic errors caused by under-recorded hearing-aid status.

The screening itself would be delivered via adult audiology services commissioned by Integrated Care Boards (ICBs), working in partnership with GP practices. Adults who do not pass the screen would move directly to a full audiological assessment, and if hearing loss is confirmed they would be offered hearing aids and follow-up support; those with severe symptoms or atypical presentations would be referred through existing ear, nose and throat (ENT) or specialist pathways in line with NICE guidance (10). The most realistic way to fund this option would be as a phased, formally evaluated pilot rather than an immediate UK-wide programme. Implementation should use a staged rollout in selected areas, ideally including places with high prevalence and unmet need (9), with invitations sent by letter, text or NHS digital channels using GP-held age registers. To minimize pressure on general practice, people with a positive screen (e.g., from self-assessment) could be booked directly into audiology, rather than being routed back through a GP appointment, which is consistent with NHS England's wider encouragement of integrated adult hearing services and local use of open-access or self-referral models (14). The pilot should collect standardized data on uptake, false positives, waiting times, hearing-aid acceptance, equity of access and patient-reported outcomes, with reporting to the ICBs and NHS England. If the pilot demonstrated acceptable feasibility and outcome gains, it could then provide the evidence base needed for broader adoption.

### 2.3 Option 2: targeted risk-based screening

An alternative policy option would be a risk-based hearing loss screening, targeting specifically adults at elevated risk of cognitive decline (particularly those with suspected/diagnosed MCI, and suspected/diagnosed dementia), among those aged 65–74 attending an NHS Health Check appointment, where dementia risk and symptoms are already discussed. Local authorities in England commission the “NHS Health Check” programme, a statutory public health intervention. It targets adults aged 40–74 years, with the aims to improve their health and wellbeing through the promotion of earlier awareness, assessment, and management of the major risk factors and conditions driving premature death, disability and health inequalities in England (15). In 2013, a mandatory dementia awareness raising component was introduced within the NHS Health Check protocol for people aged over 65 years (16).

In practice, the hearing component could be embedded into relevant NHS Health Check appointments, using a short, validated screening method such as a digits-in-noise test (17), followed by full audiological assessment for people who screen positive. This is a realistic policy option because NICE already advises referral to audiology for adults with suspected or diagnosed dementia or MCI, and recommends repeat hearing assessment every 2 years if hearing loss has not already been diagnosed. This option of the screening policy would be delivered by GP practices, practice nurses or healthcare assistants conducting relevant NHS Health Checks, and NHS audiology services, with referral into ENT when red-flag features are present (10). Funding could most plausibly come through a phased local pilot, using existing service structures rather than creating a new national programme from scratch: the screening contact itself could be incorporated into locally commissioned NHS Health Check delivery or dementia-review activity, while the confirmatory assessment and treatment phase would use existing NHS audiology capacity. That funding arrangement is partly an inference from current commissioning structures, but it is grounded in the fact that NHS Health Checks are already commissioned and delivered locally, and that audiology referral for people with dementia/MCI is already supported in national guidance. Implementation should therefore begin in a small number of high-need areas, with standardized staff training, dementia-friendly communication (18), and routine monitoring of uptake, waiting times, hearing-aid acceptance and patient-reported outcomes.

However, using NHS Health Checks as the main entry point also creates a coverage limitation. The programme is intended for adults aged 40–74 without selected pre-existing conditions. This matters for hearing-loss policy because some groups excluded from NHS Health Checks are not necessarily low risk for hearing impairment. Diabetes has been associated with a higher prevalence of hearing impairment in meta-analysis, and cardiovascular risk factors have also been associated with hearing loss in population-based studies (19, 20). A risk-based pilot should therefore likely treat NHS Health Checks as a main route into screening, but not the only route. To avoid excluding clinically important groups, implementation should also test referral prompts within long-term-condition reviews, dementia or MCI reviews, and direct audiology access pathways.

## 3 Policy options appraisal

### 3.1 Public health impact

#### 3.1.1 Current policy

Detection of adult hearing loss relies on GP presentation and referral, so its public-health reach is limited. This avoids the immediate costs of a screening programme, but it also means access depends on symptom appraisal and help-seeking, which are often delayed. In England, self-reported hearing has only limited accuracy against objective testing, so people with gradual or unrecognized loss are likely to be missed; qualitative UK evidence also shows stigma and delayed readiness to seek help (21). As a result, the benefits of the current policy are concentrated among adults who already perceive a problem and engage with services, while the burden falls disproportionately on older adults and populations in whom unmet need is less visible. Because hearing-loss prevalence varies markedly across England, an opportunistic model may also entrench geographic and social inequalities. The UK NSC itself has stated that it remains unclear how well hearing loss is currently identified and managed without screening (22).

#### 3.1.2 Option 1

An age-based programme at 60, 65 and 70 would address the problem more directly by inviting adults before they self-present, thereby increasing access to audiology and earlier hearing-aid provision. Of the three options, it has the broadest reach and therefore the greatest potential absolute public-health gain. Earlier identification could therefore improve communication, functioning and quality of life for a large age-eligible population. However, its burden would be wider too: some people would undergo assessment unnecessarily, and increased referrals could strain audiology capacity and delay care elsewhere.

#### 3.1.3 Option 2

A dementia-risk approach has narrower reach than age-based screening, but potentially greater impact per person screened because it focuses on adults with suspected or diagnosed MCI, dementia, or elevated cognitive risk, in whom hearing loss is common and especially consequential (23, 24). Public-health benefits would likely include earlier hearing assessment, better communication, and improved day-to-day functioning for patients and carers. The ACHIEVE trial further suggests that hearing intervention may slow cognitive change in older adults already at increased risk of decline (25), and the *All Wales Dementia Hearing Loss Pathway* reported 97% identification of hearing loss and 84% uptake of hearing aids among newly referred patients (26). This option is also the strongest on equity if targeted toward deprived or underserved groups, but its narrower scope means many adults with hearing loss outside dementia-risk pathways would still be missed.

### 3.2 Feasibility

#### 3.2.1 Current policy

Because of a common tendency among policy makers to reduce new (potentially) unfunded pressures, the status quo is the most politically feasible to maintain, but not necessarily easy to justify as effective. For GP self-referral, social and cultural barriers also weaken practical performance, because delayed help-seeking, stigma and limited symptom recognition mean that many adults do not enter the pathway early. Operationally, no new infrastructure is needed, of course, but the “no screening” policy's dependence on variable local practice and patient initiative makes it a weak platform for reducing unmet need.

#### 3.2.2 Option 1

An age-based screening programme would require either a national policy reversal or, more realistically, a time-limited pilot. Potential resistance would come from commissioners and providers concerned about affordability, capacity and opportunity cost. Operationally, the NHS does have some enabling infrastructure: GP records can identify eligible age cohorts, adult audiology is already a recognized community service, and NHS guidance already describes direct-access pathways. However, implementation would require new invitation systems, workforce training, increased diagnostic capacity, hearing-aid follow-up. This matters because current NHS policy is simultaneously pushing care from hospital to community and from sickness to prevention, which supports the logic of screening, but also highlights the need to avoid overloading services (27).

#### 3.2.3 Option 2

A dementia-risk-based policy is more feasible, because it can be framed as targeted case-finding within existing services rather than a new universal national screening programme. Politically, it aligns better with current guidance. The strongest operational advantage is that implementation can begin through pilots in memory clinics, dementia reviews or selected Health Check pathways, rather than requiring a full call-recall programme. The *All Wales Dementia Hearing Loss Pathway* shows that this is transferable in practice (26), although not without added staffing and service redesign. Overall, this option is flexible and more adaptable to local systems, but still needs careful piloting before wider rollout.

### 3.3 Economic and budgetary impacts

#### 3.3.1 Current policy

From a budgetary perspective, the status quo is the least expensive option to maintain. However, this apparent affordability masks a weak economic case: untreated hearing loss imposes large wider societal costs; in the UK, these are considerable, with annual economic losses estimated at £25.5 billion, comprising £9 billion in lost productivity and £16.5 billion attributable to diminished quality of life (28).

#### 3.3.2 Option 1

This is the most demanding option in budget terms, because the NHS would need to fund invitations, screening appointments, follow-up assessments, hearing aids and additional workforce capacity. Morris et al. (11) modeled the cost of this scenario, with an incremental cost-effectiveness ratio (ICER) of £1,461 per quality-adjusted life year (QALY) vs. GP referral, and 15,437 additional adults per 100,000 benefiting from hearing intervention. In economic terms, that is a very favorable result and well below usual UK willingness-to-pay thresholds, with inflation-adjusted estimates remaining comfortably within NICE limits. The main limitation is that the evidence is still driven largely by modeling; Morris et al. (11) explicitly note uncertainty around hearing-aid uptake after screening and the exclusion of some set-up costs.

#### 3.3.3 Option 2

This option is likely to offer the best budgetary compromise because it targets a smaller, higher-yield group and can use existing contacts in memory or cognitive-care pathways. NICE-based modeling summarized in Stancel-Lewis et al. (2) suggests that biannual audiology reassessment for people with MCI or dementia has an ICER of £14,337 per QALY, still within conventional UK cost-effectiveness thresholds (£20,000–£30,000). Mukadam et al. (29) modeled that screening and treating hearing loss in midlife to reduce dementia risk could even be cost saving, with a discounted lifetime saving of £607 per person and lower dementia-related care costs.

### 3.4 Policy options assessment table

The overall scores resulting from the policy analysis according to the Centers for Disease Control and Prevention (CDC) framework (30) are reported in Table 2.

**Table 2:** CDC policy analysis table for the adults' hearing loss screening options.

Criteria	Public health impact	Feasibility	Economic and budgetary impact	
			Budget	Economic
*Status quo* (no screening)	Low	Low	Less favorable	Less ***favorable***
	Medium	Medium	Favorable	Favorable
	High	High	More ***favorable***	More favorable
	Concerns about the amount or quality of data? (yes/no)	Concerns about the amount or quality of data? (yes/no)	Concerns about the amount or quality of data? (yes/no)	Concerns about the amount or quality of data? (yes/no)
*Option 1* (age-based screening)	Low	Low	Less ***favorable***	Less favorable
	Medium	Medium	Favorable	Favorable
	High	High	More favorable	More ***favorable***
	Concerns about the amount or quality of data? (yes/no)	Concerns about the amount or quality of data? (yes/no)	Concerns about the amount or quality of data? (yes/no)	Concerns about the amount or quality of data? (yes/no)
*Option 2* (risk-based screening)	Low	Low	Less favorable	Less favorable
	Medium	Medium	Favorable	***Favorable***
	High	High	More ***favorable***	More favorable
	Concerns about the amount or quality of data? (yes/no)	Concerns about the amount or quality of data? (yes/no)	Concerns about the amount or quality of data? (yes/no)	Concerns about the amount or quality of data? (yes/no)

Bold font represents the selected choice for each dimension.

## 4 Recommendations and discussion

The WHO Hearing Screening Considerations for Implementation guidance recommends regular hearing screening for older people, beginning at age 50 years, with 5-yearly screening until age 64 and screening every 1–3 years from age 65 onwards, where feasible (31). However, because the current UK NSC position does not recommend adult hearing-loss screening, this broad WHO model was seen as an international implementation benchmark rather than a directly applicable policy scenario; the options appraised here therefore focused on more targeted, pilotable routes that could be tested within existing NHS frameworks. The position taken in this policy brief is therefore not that the UK NSC was wrong to reject an immediate universal adult screening programme, but that a “do not screen” recommendation should not be interpreted as a reason to avoid targeted implementation pilots. Several of the UK NSC concerns relate to uncertainty about screening accuracy, uptake and downstream benefit; however, peer-reviewed evidence indicates that screening tools can indeed detect hearing loss in older adults, that primary-care hearing screening can improve diagnostic follow-up and hearing-aid acquisition compared with no screening, and that adult hearing-screening strategies can be cost-effective in high-income health systems (32, 39). The argument against the status quo is therefore pragmatic rather than categorical: current evidence may still be insufficient for a UK universal programme, but should be seen as strong enough to justify targeted, equity-sensitive pilots designed to generate the implementation evidence that the UK NSC identifies as missing.

Overall, Option 2, targeted risk-based screening embedded in NHS Health Checks, is the strongest recommendation. Its main limitation is that the evidence base is still developing and relies partly on modeling and pilot data. A further practical concern is that NHS Health Check uptake is itself incomplete and varies between areas, so the pathway may miss some eligible people from the outset, and mitigation actions should be put in place for this matter (33). One may also argue that dementia- or cognitive-risk-based screening is effectively pathway selection: by design, this approach prioritizes people already recognized as having cognitive symptoms, MCI, dementia, or dementia-related risk, and may therefore miss adults with clinically important hearing loss who have no cognitive diagnosis, are outside NHS Health Checks eligibility, or are not currently engaged with relevant care pathways. For this reason, targeted risk-based screening should be presented as a pragmatic first step rather than a substitute for wider adult hearing-loss case-finding. Evaluation should measure not only uptake and outcomes among those invited, but also the characteristics of groups not reached by the pathway. The proposed pilot phase of this policy option should therefore be designed around several key research questions: whether targeted hearing screening can be feasibly embedded within NHS Health Checks, dementia/MCI reviews and audiology pathways; whether it increases confirmed diagnosis, hearing-aid uptake and sustained rehabilitation engagement compared with usual care; whether uptake and benefit differ by age, deprivation, ethnicity, cognitive status and region; what additional demand it creates for audiology, primary care and follow-up services; and whether the pathway is cost-effective once screening, diagnostic assessment, hearing technologies, follow-up and rehabilitation support are considered together. These data would help determine whether later implementation requires supplementary routes, such as age-based invitations, community-based testing, or expanded self-referral into audiology. On the other hand, looking at arguments in support of this targeted approach, there is converging evidence in literature that the potential benefit of hearing intervention may be greatest among adults already at higher risk of cognitive decline: a secondary analysis of the ACHIEVE trial found that hearing intervention slowed cognitive decline most clearly in participants in the highest quartile of predicted cognitive-decline risk (34). This risk-based approach is also likely to be high-yield because population-level evidence shows that hearing loss is highly prevalent among people living with dementia, while hearing-aid use remains low, indicating a substantial unmet care gap within the very pathways proposed for targeted screening (35).

From a right-to-health perspective, regional inequalities in unmet hearing need are not only epidemiological findings but also equity concerns: the Office of the United Nations High Commissioner for Human Rights/World Health Organization framework emphasizes that health services should be available, accessible, acceptable and of good quality without discrimination, and that targeted measures may be required for groups facing particular health disadvantages (36). In the current UK policy context, a universal programme is unlikely to be adopted soon because the UK NSC still does not recommend adult hearing screening, whereas NICE already supports audiology referral and two-yearly reassessment for adults with suspected or diagnosed dementia or MCI. Compared with the status quo, Option 2 is more proactive and potentially more equitable, particularly for people at elevated dementia risk. Compared with Option 1, it is narrower in reach but more feasible, better aligned with existing pathways, and less demanding in budgetary terms. The economic case for adult hearing screening should therefore include not only the cost of screening and diagnostic assessment, but also the financing of downstream rehabilitation. The WHO guidance on rehabilitation financing emphasizes that rehabilitation should be integrated into health financing arrangements to ensure sustainable access, quality and financial protection; this is directly relevant to hearing screening, because identification without affordable access to hearing technologies, follow-up and rehabilitation support would risk widening rather than reducing unmet need (37). Universal newborn hearing screening provides a useful precedent: systematic screening can bring forward diagnosis and intervention, with evidence of improved language outcomes when permanent childhood hearing impairment is identified early (38). However, its benefits depend on timely diagnostic confirmation, follow-up and access to intervention, reinforcing that adult hearing screening should be designed as a complete care pathway rather than a standalone test.
